# Survival analysis of borderline ovarian tumors: a 23-year retrospective study in a Middle Eastern cohort

**DOI:** 10.1186/s12905-025-04183-3

**Published:** 2025-12-12

**Authors:** Haleh Ayatollahi, Sayna Abbaszadeh, Sedigheh Ghasemian Dizaj Dehr, Hamid Reza Khalkhali, Alireza Babajani

**Affiliations:** 1 Department of Obstetrics and Gynecology, School of Medicine, Urmia University of Medical Sciences, Urmia, Iran; 2 Department of Obstetrics and Gynecology, School of Medicine, Khoy University of Medical Sciences, Khoy, Iran; 3 Reproductive Health Research Center, Clinical Research Institute, Urmia University of Medical Sciences, Urmia, Iran; 4 Patient Safety Research Center, Clinical Research Institute, Urmia University of Medical Sciences, Urmia, Iran; 5 Department of Anesthesiology, School of Allied Medical Sciences, Alborz University of Medical Sciences, Karaj, Iran

**Keywords:** Borderline ovarian tumors, Hysterectomy, Survival rate, Retrospective study

## Abstract

**Background:**

Borderline ovarian tumors (BOTs) constitute a category of non-invasive epithelial neoplasms of the ovary, characterized by a substantially more favorable prognosis in comparison to their invasive malignant counterparts. Given their prevalence among young women, balancing oncologic safety with fertility preservation is critical for clinical management. This study evaluates factors associated with survival in patients with BOTs over a long-term follow-up period.

**Materials and methods:**

This retrospective cohort study examined data from 135 patients diagnosed with borderline ovarian tumors (BOTs) between January 2000 and December 2023 at medical centers associated with Urmia University of Medical Sciences. Clinical and histopathological parameters were carefully extracted from both electronic and paper-based medical records. Overall survival rates were estimated using the Kaplan-Meier method, and intergroup comparisons were performed with the log-rank test.

**Results:**

The mean patient age was 39.5 years; with most cases diagnosed at FIGO stage I (94.1%). Conservative surgical management was performed in 65.2% of patients, while 34.8% underwent total abdominal hysterectomy with bilateral salpingo-oophorectomy (TAH + BSO). Survival rates at 1, 3, 5, and 10 years were 100%, 99.2%, 98.3%, and 97.2%, respectively. No statistically significant differences in survival were observed based on age) *p* = 0.442(, tumor size (*P* = 0.752), bilaterality (*P* = 0.969), Histopathology (*P* = 0.069), FIGO stage (*P* = 0.746), or surgical approach (*P* = 0.199).

**Conclusion:**

Borderline ovarian tumors (BOTs) typically manifest a benign clinical trajectory, with conservative surgical approaches demonstrating both safety and efficacy, especially in women of reproductive age. The absence of correlations between select histopathological characteristics and survival outcomes underscores the need to avoid unnecessary surgical procedures, thereby optimizing patient-centered care.

## Background

Borderline ovarian tumors (BOTs), also known as low malignant potential tumors, are a histologically heterogeneous subset of epithelial ovarian neoplasms characterized by atypical epithelial proliferation without stromal invasion [1]. BOTs constitute less than 20% of all ovarian tumors and are predominantly serous or mucinous in histology. Unlike invasive epithelial ovarian cancers, BOTs exhibit a relatively indolent clinical course and a more favorable prognosis [2]. Because these tumors are often diagnosed in women of reproductive age, clinical management requires careful consideration of both oncologic safety and fertility preservation [2, 3].

Borderline ovarian tumors are most frequently identified at an early stage, with epidemiological evidence showing that nearly 70% of cases present at FIGO stage I, followed by 10% at stage II, 19% at stage III, and only 1% at stage IV [4]. The clinical outcome of affected patients is influenced by several key factors, including the stage of disease at diagnosis, histopathological subtype, reproductive characteristics (such as parity and menopausal status), and the extent and type of surgical management performed [5]. BOTs are generally associated with favorable long-term outcomes. Among women diagnosed at FIGO stages I–III, the five-year survival rate is approximately 95%, and the ten-year survival rate remains close to 90%, underscoring the excellent prognosis observed in early to intermediate stages of the disease. In contrast, patients presenting with FIGO stage IV BOTs exhibit a markedly reduced survival rate of about 77%, reflecting the adverse impact of advanced disease and metastatic dissemination. These findings underscore the prognostic relevance of disease stage at diagnosis and highlight the critical importance of early detection and appropriate clinical management in optimizing patient outcomes [6].

The extant literature on borderline ovarian tumors (BOTs) has predominantly concentrated on isolated prognostic determinants. Although some studies have delved into surgical approaches and survival outcomes [7], others have centered on non-invasive peritoneal implants [8], histopathological subtypes [9, 10], or reproductive covariates, including parity and fertility aspirations [11]. However, few studies have provided a comprehensive survival analysis that integrates demographic, reproductive, and treatment-related variables. The present retrospective investigation seeks to redress this shortfall by proffering a multifaceted appraisal of prognostic determinants for short-term (1- and 3-year) and long-term (5- and 10-year) survival among women diagnosed with borderline ovarian tumors (BOTs) between 2000 and 2023.

## Methods

### Study design

This retrospective study included patients diagnosed with borderline ovarian tumors (BOTs) who underwent treatment at hospitals affiliated with Urmia University of Medical Sciences between 2000 and 2023. The study protocol was approved by the Institutional Ethics Committee for Human Research at Urmia University of Medical Sciences, Ethics Code IR UMSU REC 1401 119, and in accordance with the principles of the Declaration of Helsinki.

The research team reviewed surgical pathology records from oncology departments of hospitals affiliated with Urmia University of Medical Sciences. Patients were identified based on clinical documentation, pathology slides, and histopathological reports verified by the pathologist. Initially, 183 cases were identified, of which 48 patients were excluded from the study process due to incomplete clinical data and unavailability of follow-up information. As a result, 135 patients were included in the final analysis.

### Data collection and statistical analysis

It is noteworthy that during the preoperative evaluation of patients with suspected borderline ovarian tumors, in planning the surgical approach, the research team examined the patients’ history of oral contraceptive pill use and assessed their fertility status before surgery (Table 1).

**Table 1:** Characteristics of the study population (*n* = 135)

Variable	Mean ± SD, Frequency (%)	Median (IQR)
**Age (years)**	39.50 ± 12.24	37 (31–48)
**Tumor size (cm)**	12.70 ± 7.17	12 (7–15)
**Number of pregnancies**	2.44 ± 2.35	2 (0–3)
Age group		
< 30 years	30 (22.2%)	
30–50 years	75 (55.6%)	
>50 years	30 (22.2%)	
Menopausal status		
No	115 (85.2%)	
Yes	20 (14.8%)	
History of OCP use		
No	126 (93.3%)	
Yes	9 (6.7%)	
Number of pregnancies		
None	35 (25.9%)	
1–2	51 (37.8%)	
≥ 3	49 (36.3%)	
Initial clinical complaint		
Abdominal pain	101 (74.8%)	
Abdominal distention	17 (12.6%)	
AUB/PMB	17 (12.6%)	
Tumor location		
Unilateral	114 (84.4%)	
Bilateral	21 (15.6%)	
Tumor size category		
< 10 cm	50 (37.0%)	
10–20 cm	66 (48.9%)	
>20 cm	19 (14.1%)	
Histopathology		
Serous	78 (57.8%)	
Mucinous	56 (41.5%)	
Endometriosis	1 (0.7%)	
FIGO Stage		
FIGO 1	127 (94.1%)	
FIGO 2	4 (3.0%)	
FIGO 3	4 (3.0%)	
Type of surgery		
TAH + BSO	47 (34.8%)	
Conservative surgery	88 (65.2%)	
Recurrence		
No	113 (83.7%)	
Yes	22 (16.3%)	
Micro invasion		
No	119 (88.1%)	
Yes	16 (11.9%)	
Outcome		
Alive	132 (97.8%)	
Death	3 (2.2%)	

Continuous variables are presented as Mean ± SD and Median (IQR: Interquartile Range). Categorical variables are presented as Frequency (Percent)

*OCP* Oral Contraceptive Pill, *AUB* Abnormal Uterine Bleeding, *PMB* Postmenopausal Bleeding, *TAH + BSO* Total Abdominal Hysterectomy with Bilateral Salpingo-Oophorectomy

We collected data for survival analysis using a checklist developed by the principal investigator. Participants were followed longitudinally for a follow-up period ranging from 15 to 237 months, with a mean follow-up period of 99.05 ± 54.2 months (from January 2000 to December 2023). Post-diagnosis surveillance for each patient was performed according to a structured protocol that included assessments at 6-month intervals during the initial 2 years and annual evaluations thereafter. The final review date for the survival assessment was December 2023.

The investigated variables encompassed demographic features, including age at diagnosis and age grouping; clinical attributes, such as history of infertility, parity (number of deliveries), tumor dimensions, tumor site, histological tumor classification, and FIGO staging; as well as treatment-associated factors, comprising histopathological subtype and modality of surgical procedure.

In accordance with the statistical consultant’s recommendation and owing to the limited number of observed events (*n* = 3 deaths), multivariate Cox regression was not employed to prevent model overfitting and the generation of unreliable parameter estimates. Therefore, Survival was evaluated using univariate Kaplan-Meier analysis and log-rank tests. All statistical analyses were performed with 95% confidence intervals, and *p* < 0.05 was considered statistically significant.

## Results

### Study population

**Fig. 1:**
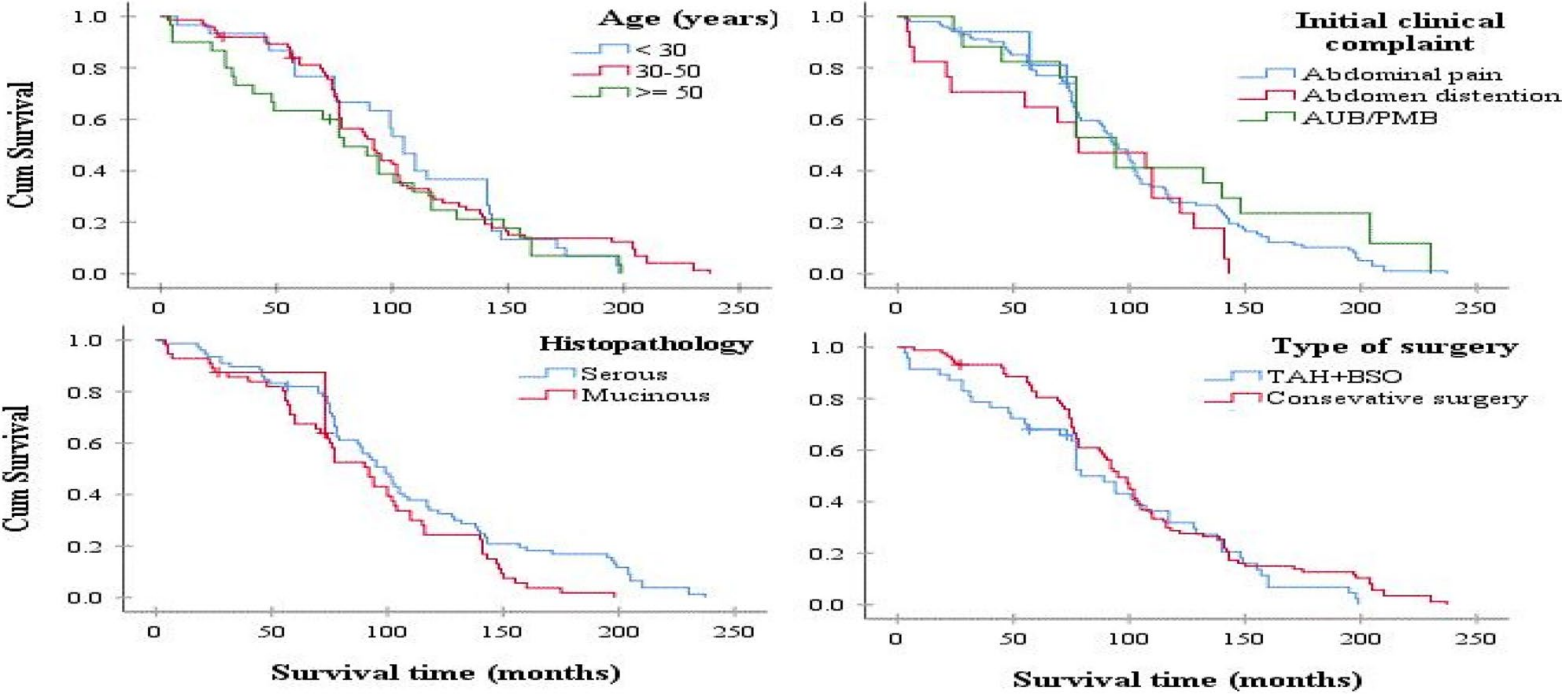
Kaplan-Meier Survival Curves by Age Group, Histopathological Subtype, Initial Clinical Complaint, and Type of Surgery

The study initially enrolled 183 women who underwent surgery for borderline ovarian tumors (BOTs) between January 2000 and January 2023. Forty-eight patients were excluded due to incomplete clinical data and unavailability of follow-up information, leaving 135 patients with confirmed BOT diagnoses based on histopathology. Patients were classified by FIGO stage and tumor characteristics, as shown in Fig. 1. Their ages ranged from 16 to 78 years (mean 39.5 ± 12.2 years). At diagnosis, most patients (85.2%) were in their reproductive years and premenopausal, while 14.8% were postmenopausal. Additionally, 6.7% reported long-term use of oral contraceptive pills (OCPs). Regarding pregnancy history, 25.9% had never been pregnant, 37.8% had one or two pregnancies, and 36.3% had three or more pregnancies.

The clinical presentation was predominantly abdominal pain (74.8%), while smaller proportions presented with abdominal distention (12.6%) or abnormal uterine bleeding/postmenopausal bleeding (12.6%). Histopathological findings indicated that serous tumors were the most common (57.8%), followed by mucinous tumors (41.5%). Only one case (0.7%) was an endometrioid tumor. Most patients (84.4%) had unilateral tumors, with bilateral involvement in 15.6% of cases. The complete details regarding tumor type and laterality are presented in Table 2.

**Table 2:** Tumor type and laterality characteristics

Histological Type	Unilateral (*n*, %)	Bilateral (*n*, %)	Total (*n*)
Serous	66 (84.6%)	12 (15.4%)	78
Mucinous	47 (83.9%)	9 (16.1%)	56
Endometrioid	1 (100%)	0 (0%)	1
Total	114 (84.4%)	21 (15.6%)	135

It is imperative to underscore that all patients with clinical suspicion of borderline ovarian tumors underwent a meticulous preoperative staging protocol. This exhaustive assessment encompassed biochemical tumor marker profiling, chest radiography, meticulous abdominal palpation, comprehensive pelvic examination, and, in select cases, computed tomography (CT) and magnetic resonance imaging (MRI) scans. Based on the findings of these evaluations, the patients were subsequently deemed suitable candidates for surgical intervention. During the operative phase, we performed cytological evaluation of peritoneal fluid and carefully examined the abdominal cavity for metastatic lesions. In patients whose histopathological findings confirmed mucinous histology, we also performed appendectomy. Pathological evaluation showed the absence of lymph node metastasis in all cases, thus eliminating the need for lymphadenectomy. Furthermore, omental biopsies were harvested from every participant.

According to FIGO staging, the majority of patients (94%) were diagnosed at stage I, and only a small proportion were diagnosed at stages II and III, each accounting for 3% of cases. In terms of surgical treatment, 65.2% of patients underwent conservative surgery to preserve fertility, such as cystectomy or unilateral oophorectomy, while 34.8% underwent total abdominal hysterectomy with bilateral salpingo-oophorectomy (TAH + BSO). According to the follow-up after conservative surgery, four patients became pregnant successfully, one of whom experienced a triplet pregnancy.

Regarding lymph node assessment, all patients were stratified according to the FIGO classification system based on histopathological findings. In the absence of identifiable risk factors and among those with early-stage disease (i.e., FIGO stage I), lymphadenectomy was not performed. In contrast, patients with advanced FIGO stage or intraoperative suspicion of nodal involvement underwent comprehensive lymph node evaluation. Notably, pathological examination revealed no lymph node metastases in any of the cases assessed.

Microscopically, micro invasion was reported in 11.9% of cases, while it was absent in 88.1%. The mean tumor size was 13.37 ± 7.02 cm, with sizes ranging from 3 to 40 cm. Tumor size distribution showed that 48.9% of tumors measured between 10 and 20 cm, 37% were smaller than 10 cm, and 14.1% exceeded 20 cm (Table 1).

The patients were followed for a period ranging from 15 to 237 months, with a mean follow-up duration of 99.05 ± 54.2 months. During the follow-up period and the course of the study (from 2000 to 2023), only 3 out of 135 patients (2.2%) had died. The overall survival function was estimated using the Kaplan–Meier method. The mean overall survival time was 231.9 ± 2.8 months (95% CI). The 1-, 3-, 5-, and 10-year survival rates were reported as 100%, 99.2%, 98.3%, and 97.2%.

The average survival time for patients aged under 30, 30–50, and over 50 years was 106.3 ± 8.9 months (95% CI: 88.8–123.9), 103.8 ± 6.4 months (95% CI: 91.3–116.4), and 86.2 ± 10.5 months (95% CI: 69.2–109.7), respectively. According to the Log-rank test, there was no statistically significant difference in survival among the different age groups (*P* = 0.44), as shown in Fig. 1.

**Fig. 2:**
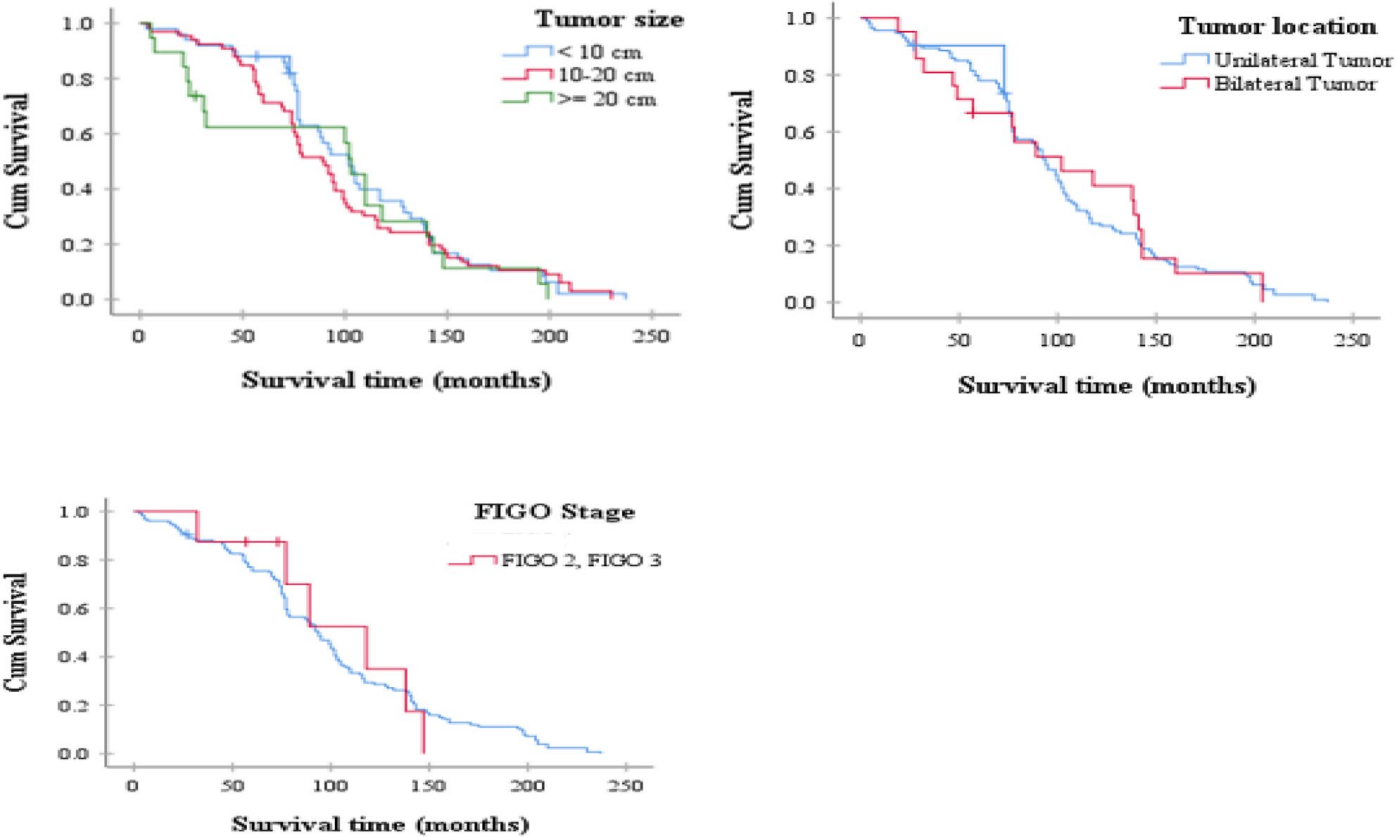
Kaplan-Meier Survival Curves by Tumor size, Tumor location, FIGO Stage

The average survival time for patients with tumors larger than 20 cm was 90.9 ± 14.7 months (95% CI: 62.1–119.8), which was shorter compared to patients with tumors smaller than 10 cm (107.8 ± 4.7 months, 95% CI: 93.3–126.4) and those with tumors measuring between 10 and 20 cm (98.8 ± 4.6 months, 95% CI: 83.4–111.4). According to the results of the Log-rank test, there was no statistically significant difference in survival based on tumor size (*P* = 0.752), as shown in Fig. 2.

The average survival time for patients with unilateral tumors was 100.4 ± 7.5 months (95% CI: 62.1–119.8). In comparison, patients with bilateral borderline ovarian tumors had a mean survival time of 99.9 ± 7.7 months (95% CI: 76.1–126.8). However, the Log-rank test indicated that this difference was not statistically significant (*P* = 0.969), as shown in Fig. 2.

The overall survival of patients with borderline ovarian tumors at FIGO stages I to III showed no statistically significant difference between the three groups based on the Log-rank test (*P* = 0.746). However, due to the limited number of patients in stage III, an accurate assessment for this group was not feasible, as shown in Fig. 2.

The average survival time for patients who underwent TAH + BSO surgery was 91.4 ± 1.8 months (95% CI: 75.3–107.3), while for those who underwent conservative surgery it was 105.4 ± 4.5 months (95% CI: 94.1–116.8). Although survival was longer in the conservative surgery group, this difference was not statistically significant based on the Log-rank test (*P* = 0.199), as shown in Fig. 1.

The average survival time for patients with serous tumors was 108.5 ± 5.6 months (95% CI: 95.3–121.3), while for those with mucinous tumors it was 89.4 ± 7.6 months (95% CI: 77.1–101.8). Although patients with serous tumors had a longer survival, this difference was not statistically significant according to the Log-rank test (*P* = 0.069), as shown in Fig. 1. General information regarding the obtained results is also presented in Table 3.

**Table 3:** Survival analysis of patients with borderline ovarian tumors by Demographic, Clinical, and pathological characteristics

Variable & Category	Mean Survival (95% CI) (months)	Median Survival (IQR) (months)	Log-Rank Test
Age (years)			χ² (2) = 1.632, *p* = 0.442
< 30	106.4 (88.8–123.9.8.9)	105.0 (75.0–142.0.0.0)	
30–50	103.9 (91.3–116.4.3.4)	92.0 (74.0–132.0.0.0)	
>50	86.3 (65.7–106.9.7.9)	79.0 (32.0–117.0.0.0)	
Initial clinical complaint			χ² (2) = 3.761, *p* = 0.153
Abdominal pain	101.7 (91.5–111.9.5.9)	95.0 (71.0–139.0.0.0)	
Abdominal distention	78.9 (54.5–103.3.5.3)	78.0 (23.0–122.0.0.0)	
AUB/PMB	114.8 (82.5–147.0)	94.0 (77.0–148.0.0.0)	
Tumor location			χ² (1) = 0.002, *p* = 0.969
Unilateral	100.5 (90.5–110.4.5.4)	94.0 (71.0–132.0.0.0)	
Bilateral	101.4 (76.4–126.4.4.4)	102.0 (49.0–143.0.0.0)	
Tumor size			χ² (1) = 0.571, *p* = 0.752
< 10 cm	107.5 (93.1–121.8.1.8)	102.0 (77.0–140.0.0.0)	
10–20 cm	98.1 (85.0–111.1.0.1)	89.0 (58.0–122.0.0.0)	
>20 cm	91.0 (62.2–119.8.2.8)	103.0 (24.0–140.0.0.0)	
Histopathology			χ² (1) = 4.840, *p* = 0.028
Serous	108.6 (95.7–121.5.7.5)	99.0 (75.0–140.0.0.0)	
Mucinous	89.5 (77.0–102.0.0.0)	92.0 (57.0–116.0.0.0)	
FIGO Stage			χ² (1) = 0.000, *p* = 0.995
FIGO 1	100.2 (90.6–109.8.6.8)	94.0 (69.0–140.0.0.0)	
FIGO 2&3	103.6 (72.9–134.2.9.2)	118.0 (77.0–138.0.0.0)	
Type of surgery			χ² (1) = 1.646, *p* = 0.199
TAH + BSO	91.4 (75.4–107.4.4.4)	79.0 (48.0–138.0.0.0)	
Conservative	105.4 (94.2–116.6.2.6)	95.0 (74.0–141.0.0.0)	
Menopausal status			χ² (1) = 1.300, *p* = 0.254
No	103.4 (93.5–113.2.5.2)	94.0 (74.0–140.0.0.0)	
Yes	84.3 (59.0–109.6.0.6)	79.0 (28.0–129.0.0.0)	
History of OCP use			χ² (1) = 0.100, *p* = 0.751
No	100.3 (90.6–110.1.6.1)	94.0 (60.0–140.0.0.0)	
Yes	100.8 (75.9–125.6.9.6)	78.0 (72.0–117.0.0.0)	
Number of pregnancies			χ² (2) = 0.490, *p* = 0.783
None	98.7 (80.2–117.2.2.2)	92.0 (58.0–140.0.0.0)	
1–2	97.7 (82.8–112.6.8.6)	78.0 (60.0–132.0.0.0)	
≥ 3	104.7 (89.3–120.2.3.2)	102.0 (77.0–140.0.0.0)	
Micro invasion			χ² (1) = 0.004, *p* = 0.950
No	99.3 (89.2–109.5.2.5)	94.0 (58.0–139.0.0.0)	
Yes	108.8 (91.1–126.4.1.4)	90.0 (77.0–140.0.0.0)	

Due to the limited number of patients with FIGO stage II (n=4) and FIGO stage III (n=4), these categories were combined into a single group (FIGO II-III) for the survival and regression analyses to ensure more reliable and stable estimates. Furthermore, the single case with endometriosis histology was excluded from the survival and regression analyses to prevent potential bias and overfitting caused by this very small subgroup

*CI* Confidence Interval, *IQR* Interquartile Range, *AUB* Abnormal Uterine Bleeding, *PMB* Postmenopausal Bleeding, *OCP* Oral Contraceptive Pill, *TAH + BSO* Total Abdominal Hysterectomy with Bilateral Salpingo-Oophorectomy

## Discussion

Borderline ovarian tumors (BOTs) are a heterogeneous group of ovarian lesions characterized by atypical epithelial proliferation without stromal invasion [1]. Their prognosis primarily depends on the stage at diagnosis and histopathological features, with most studies reporting favorable outcomes [7]. BOTs are frequently diagnosed at early stages, contributing to high survival rates. However, like invasive ovarian carcinomas, BOTs can spread to the peritoneum and lymph nodes, necessitating the identification of risk factors for aggressive recurrence or disease-related mortality [8, 12].

This retrospective cohort study evaluated survival rates and their associated determinants in a cohort of 135 patients diagnosed with borderline ovarian tumors (BOTs)، and referred to the Gynecologic Oncology Department at Urmia University of Medical Sciences between January 2000 and December 2023. Participants were prospectively monitored for a follow-up period ranging from 15 to 237 months (mean 99.05 ± 54.2 months). One of the principal methodological strengths of this investigation lies in its multivariate analysis of survival outcomes, stratified by demographic, reproductive, and therapeutic covariates, underpinned by an extended observational period that affords a comprehensive evaluation of long-term clinical trajectories. Furthermore, within the Middle Eastern region, retrospective studies encompassing an approximate 23-year follow-up duration and a cohort of 135 patients remain exceedingly scarce.

Our findings indicate that most BOTs were serous (57.8%), followed by mucinous (41.5%), with the majority being unilateral and diagnosed at FIGO stage I. These results align with studies identifying serous BOTs as the most common subtype [9, 11]. In contrast, a large cohort study by Karlsen et al. reported nearly equal prevalence of serous and mucinous BOTs [13]. while other studies noted a higher prevalence of mucinous BOTs [14], These variations may be attributed to geopolitical and demographic factors, including ethnicity, environmental exposures, and lifestyle differences. Notably, serous BOTs appear to be more prevalent in Middle Eastern populations, whereas mucinous BOTs are more commonly observed in East Asian populations [15]. The near-total absence of the endometrioid subtype of borderline ovarian tumors (endometrioid BOTs) observed in the present cohort may underscore region-specific epidemiological idiosyncrasies. This observation aligns with corroborative evidence from select investigations. It merits emphasis that, notwithstanding their infrequency, the discernment of endometrioid BOTs assumes paramount clinical significance, given their propensity to evolve into low-grade endometrioid carcinoma [16].

Following fertility assessment after conservative surgical intervention, four patients achieved successful pregnancies, with one patient experiencing three such outcomes. It merits emphasis that the study was conducted in a demographic context characterized by early marital unions and childbearing among women. Considering that the majority of participants were approximately 35 years of age, the reported number of pregnancies is relatively low.

We found that patients with serous borderline ovarian tumors had higher survival rates than those with mucinous BOTs, though this difference was not statistically significant(*P* = 0.069). These findings align with some studies [17, 18], while others report a significant difference [16], possibly due to variations in disease stage across study populations. Additionally, certain studies suggest that mucinous BOTs have a greater potential for malignant transformation and progression to invasive carcinoma [19, 20].Our study results demonstrate that BOTs are distinct from invasive ovarian cancers. The lack of significant survival differences based on clinic pathological factors, such as tumor size(*P* = 0.752) or bilaterality (*P* = 0.969), suggests that molecular and genetic features may be more reliable predictors of recurrence or survival [21].

Our study results showed that traditional clinic pathological factors have limited predictive value for survival in borderline ovarian tumors (BOTs). Instead, molecular profiles provide a more accurate assessment of biological behavior. Recent studies have identified frequent mutations in the KRAS, BRAF, and ERBB2 genes in serous and mucinous BOTs. These mutations affect the MAPK signaling pathway and influence tumor pathogenesis and clinical outcomes [22, 23].

Overall survival among patients with BOTs at FIGO stages I to III showed no statistically significant differences (*P* = 0.746). This contrasts with studies reporting lower 5-year survival rates with advancing disease stage, typically due to increased mortality risk at higher stages [13, 24]. A possible explanation for the lack of a significant association in our study is that the majority of patients were diagnosed at stage I, while the number of cases identified at stage III was too small to yield reliable comparisons. This limited representation of advanced-stage cases is considered one of the main limitations of our study.

In the present study, the mean patient age was approximately 40 years, with the majority of participants aged between 30 and 50 years; nonetheless, a wide age distribution was evident. This finding is consistent with reports from select prior investigations [11, 24]. Conversely, certain studies have documented a higher mean age at diagnosis relative to the current cohort [13, 25], whereas others have indicated a lower mean age [14]. These inconsistencies may be attributable to differences in participant recruitment strategies and sociocultural variations in healthcare utilization patterns, particularly those influencing the timing of ovarian tumor detection. Notably, no substantial disparities in survival outcomes were identified across age strata in this analysis(*P* = 0.44). Such results diverge from those of other studies [3, 17], which may be explained by the age-related propensity for malignant progression in recurrent borderline ovarian tumors.

No significant disparity in median survival rates was observed between patients undergoing total abdominal hysterectomy with bilateral salpingo-oophorectomy (TAH + BSO) and those receiving conservative surgical management(*P* = 0.199). This observation aligns with results from select prior investigations [9, 26]. For instance, Mencher et al. examined 225 patients with borderline ovarian tumors, including 147 with FIGO stages II–III who underwent hysterectomy, and similarly reported no substantial difference in overall survival [26]. These results challenge the imperative role of hysterectomy in treating advanced-stage borderline ovarian tumors, positioning conservative surgery as a viable alternative for women desiring fertility preservation [27].

### Limitation

The retrospective nature of this study represents a principal limitation. The exclusion of 48 patients due to incomplete data may have engendered selection bias, thereby potentially compromising the generalizability of the findings. To counteract this, all accessible cases with comprehensive long-term follow-up were incorporated. The predominance of Stage I disease (94%) within the cohort—aligning with the characteristic epidemiological profile of borderline ovarian tumors—impeded robust statistical analyses of survival outcomes in advanced stages. Consequently, inferences regarding the relationship between disease stage and survival warrant prudent consideration, owing to the paucity of Stage II and III cases.

Future research should focus on multicenter, prospective studies with larger sample sizes to improve the generalizability of results. Additionally, parallel studies could investigate the psychological aspects and quality of life associated with patient outcomes.

## Conclusion

The findings of this study underscore that patients with borderline ovarian tumors, especially those identified at early stages and managed via conservative surgery, manifest exceptionally favorable survival outcomes. The lack of substantial disparities in survival across variables such as age, tumor size, bilaterality, or FIGO stage suggests that conventional histopathological parameters may hold limited prognostic value in this population. Furthermore, the comparable survival rates observed between patients receiving total abdominal hysterectomy with bilateral salpingo-oophorectomy (TAH + BSO) and those undergoing conservative procedures affirm the viability and safety of fertility-preserving interventions, particularly among younger women. Survival outcomes underscore the need to avoid unnecessary surgical procedures, thereby optimizing patient-centered care.

## Data Availability

Research data are available upon formal request and a privacy statement from the corresponding author.

## Abbreviations

BOTs: Borderline ovarian tumors
BSO: Bilateral Salpingo-oophorectomy
TAH: Total Abdominal Hysterectomy

## References

[1] Ricotta G, Maulard A, Genestie C, Pautier P, Leary A, Chargari C, et al. Brenner borderline ovarian tumor: a case series and literature review. Ann Surg Oncol. 2021;28(11):6714–20. 33768396 10.1245/s10434-021-09879-y

[2] du Bois A, Ewald-Riegler N, de Gregorio N, Reuss A, Mahner S, Fotopoulou C, et al. Borderline tumours of the ovary: a cohort study of the Arbeitsgmeinschaft Gynäkologische Onkologie (AGO) Study Group. Eur J Cancer. 2013;49(8):1905–14. 23490647 10.1016/j.ejca.2013.01.035

[3] Trillsch F, Mahner S, Woelber L, Vettorazzi E, Reuss A, Ewald-Riegler N, et al. Age-dependent differences in borderline ovarian tumours (BOT) regarding clinical characteristics and outcome: results from a sub-analysis of the Arbeitsgemeinschaft Gynaekologische Onkologie (AGO) ROBOT study. Ann Oncol. 2014;25(7):1320–7. 24618151 10.1093/annonc/mdu119

[4] Harter P, Gershenson D, Lhomme C, Lecuru F, Ledermann J, Provencher DM, et al. Gynecologic cancer intergroup (GCIG) consensus review for ovarian tumors of low malignant potential (borderline ovarian tumors). Int J Gynecol Cancer. 2014;24(9 Suppl 3):S5–8. 25341581 10.1097/IGC.0000000000000282

[5] Plett H, Harter P, Ataseven B, Heitz F, Prader S, Schneider S, et al. Fertility-sparing surgery and reproductive-outcomes in patients with borderline ovarian tumors. Gynecol Oncol. 2020;157(2):411–7. 32115229 10.1016/j.ygyno.2020.02.007

[6] Huchon C, Bourdel N, Abdel Wahab C, Azaïs H, Bendifallah S, Bolze PA, et al. Borderline ovarian tumors: French guidelines from the CNGOF. Part 1. Epidemiology, biopathology, imaging and biomarkers. J Gynecol Obstet Hum Reprod. 2021;50(1):101965. 33160106 10.1016/j.jogoh.2020.101965

[7] Trimble CL, Kosary C, Trimble EL. Long-term survival and patterns of care in women with ovarian tumors of low malignant potential. Gynecol Oncol. 2002;86(1):34–7. 12079297 10.1006/gyno.2002.6711

[8] Sun Y, Xu J, Jia X. The diagnosis, treatment, prognosis and molecular pathology of borderline ovarian tumors: current status and perspectives. Cancer Manage Res. 2020;12:3651–9. 10.2147/CMAR.S250394 PMC7246309 32547202

[9] Sharami SRY, Farhadifar F, Tabatabaei R. Recurrence and 5-year survival rate in patients with borderline ovarian tumors and related factors in Kurdistan. Eur J Transl Myol. 2022;33(1):10779. 36173319 10.4081/ejtm.2022.10779 PMC10141740

[10] Scaglione G, Travaglino A, Raffone A, Santoro A, Arciuolo D, Fulgione C, et al. Micropapillary pattern in serous borderline ovarian tumor and the risk of extraovarian localization of low-grade serous carcinoma (‘invasive implants’): a systematic review and meta-analysis. Pathology. 2024;264:155714. 10.1016/j.prp.2024.155714 39520971

[11] Karimi Zarchi M, Mehdizadeh Kashi A, Allahqoli L, Sadat Tabatabai R, Shamsi F, Hashemian Asl N. The recurrence and 5-year survival rates in patients with borderline ovarian tumors in Yazd from 2006 to 2016. J Obstet Gynecol Cancer Res. 2022;4(2):57–61.

[12] Pecorino B, Laganà AS, Mereu L, Ferrara M, Carrara G, Etrusco A, et al. Evaluation of borderline ovarian tumor recurrence rate after surgery with or without fertility-sparing approach: results of a retrospective analysis. Healthcare. 2023;11(13):1922. 37444757 10.3390/healthcare11131922 PMC10341047

[13] Karlsen NMS, Karlsen MA, Høgdall E, Nedergaard L, Christensen IJ, Høgdall C. Relapse and disease specific survival in 1143 Danish women diagnosed with borderline ovarian tumours (BOT). Gynecol Oncol. 2016;142(1):50–3. 27168006 10.1016/j.ygyno.2016.05.005

[14] Johansen G, Dahm-Kähler P, Staf C, Rådestad AF, Rodriguez-Wallberg KA. Reproductive and obstetrical outcomes with the overall survival of fertile-age women treated with fertility-sparing surgery for borderline ovarian tumors in Sweden: a prospective nationwide population-based study. Fertil Steril. 2021;115(1):157–63. 32977941 10.1016/j.fertnstert.2020.07.043

[15] Song T, Lee YY, Choi CH, Kim TJ, Lee JW, Bae DS, et al. Histologic distribution of borderline ovarian tumors worldwide: a systematic review. J Gynecol Oncol. 2013;24(1):44–51. 23346313 10.3802/jgo.2013.24.1.44 PMC3549507

[16] Fischerova D, Zikan M, Dundr P, Cibula D. Diagnosis, treatment, and follow-up of borderline ovarian tumors. Oncologist. 2012;17(12):1515–33. 23024155 10.1634/theoncologist.2012-0139 PMC3528384

[17] Kalapotharakos G, Högberg T, Bergfeldt K, Borgfeldt C. Long-term survival in women with borderline ovarian tumors: a population-based survey of borderline ovarian tumors in Sweden 1960–2007. Acta Obstet Gynecol Scand. 2016;95(4):473–9. 26714557 10.1111/aogs.12846

[18] Song T, Lee Y-Y, Choi CH, Kim T-J, Lee J-W, Kim B-G, et al. Prognosis in patients with serous and mucinous stage I borderline ovarian tumors. Int J Gynecol Cancer. 2012;22(5):770–7. 22426410 10.1097/IGC.0b013e31824b4076

[19] Lalwani N, Shanbhogue AK, Vikram R, Nagar A, Jagirdar J, Prasad SR. Current update on borderline ovarian neoplasms. AJR Am J Roentgenol. 2010;194(2):330–6. 20093592 10.2214/AJR.09.3936

[20] Bonadio RC, de Siqueira Santos AG, Estevez-Diz MDP. Borderline ovarian tumors: a review of its biology, molecular profile, and management. Brazilian J Oncol. 2024;20:e–20230437.

[21] Silva EG, Gershenson DM, Malpica A, Deavers M. The recurrence and the overall survival rates of ovarian serous borderline neoplasms with noninvasive implants is time dependent. Am J Surg Pathol. 2006;30(11):1367–71. 17063075 10.1097/01.pas.0000213294.81154.95

[22] Atallah D, El Feghaly I, Choueiry E, Jalkh N, Khaddage A, Akiki M, et al. 618 Genetic profile by whole exome sequencing of borderline ovarian tumors: series of 32 patients.International Journal of Gynecological Cancer. 2021;31:A256–A7.

[23] Badlaeva A, Tregubova A, Palicelli A, Asaturova A. Eosinophilic cells in ovarian borderline serous tumors as a predictor of BRAF mutation. Cancers. 2024;16(13):2322. 39001384 10.3390/cancers16132322 PMC11240704

[24] Loizzi V, Selvaggi L, Leone L, Latorre D, Scardigno D, Magazzino F, et al. Borderline epithelial tumors of the ovary: experience of 55 patients. Oncol Lett. 2015;9(2):912–4. 25621067 10.3892/ol.2014.2758 PMC4301501

[25] Sahin F, Aktürk E, Günkaya OS, Özdemir S, Konal M, Genç S, et al. Borderline ovarian tumors: twenty years of experience at a tertiary center. Anatol Curr Med J. 2023;5(3):196–200.

[26] Menczer J, Chetrit A, Sadetzki S. The effect of hysterectomy on survival of patients with borderline ovarian tumors. Gynecol Oncol. 2012;125(2):372–5. 22366596 10.1016/j.ygyno.2012.02.017

[27] Pecorino B, Laganà AS, Mereu L, Ferrara M, Carrara G, Etrusco A, et al. Evaluation of borderline ovarian tumor recurrence rate after surgery with or without fertility-sparing approach: results of a retrospective analysis. Healthcare. 2023. 10.3390/healthcare11131922. 37444757 10.3390/healthcare11131922 PMC10341047

